# Developing an AI platform for voice-input medical records in Polish: a digital application for optimisation of physicians’ workload

**DOI:** 10.1038/s41598-026-50490-6

**Published:** 2026-05-11

**Authors:** Dariusz Szplit, Andrzej Czyżewski, Beata Graff, Anna Szyndler, Piotr Pałczyński, Mariusz Siemiński, Natalia Glaner, Tomasz Stefaniak, Julia Bogdan, Kinga Słomińska, Anna Dąbkowska, Mariusz Budzisz, Marta Zielonka, Daniel Cieślak, Szymon Zaporowski, Józef Kotus, Krzysztof Narkiewicz

**Affiliations:** 1 Department of Health Care Quality, Medical University of Gdansk, M. Skłodowskiej-Curie 3a, 80-210 Gdańsk, Poland; 2 Faculty of Electronics, Telecommunications, and Informatics, Gdańsk University of Technology, Gdańsk, Poland; 3 Department of Hypertension and Diabetology, Medical University of Gdańsk, Gdańsk, Poland; 4 Department of Emergency Medicine, Medical University of Gdansk, Gdańsk, Poland; 5 University Clinical Center, Gdańsk, Poland; 6 eTrust Medical Ltd., Reda, Poland; 7 Student Scientific Circle of Emergency Medicine, Medical University of Gdansk, Gdańsk, Poland

**Keywords:** Medical text annotation, AI in healthcare, Medical speech recognition, Polish medical language, EHR improvement by AI, AI for real world data collection, AI towards quality and safety of health care, Health care, Mathematics and computing

## Abstract

In a scientific and implementation consortium, we developed an adaptive AI platform that enables doctors to create accurate and comprehensive Electronic Health Records (EHRs) through advanced speech recognition and context analysis tailored to Polish medical language. This system ensures stability with consistent performance across real-world clinical settings, achieving expected values for speech and context recognition during extensive testing. Its robustness is demonstrated by handling diverse inputs—such as regional accents, complex terminology, and noisy environments—supported by error-correction mechanisms and a specialized acoustic probe. Sustainability is achieved through seamless integration with existing healthcare infrastructures, scalable design, and ongoing updates to medical dictionaries, facilitating long-term use and adaptation. Structured data from electronic health records (EHRs) supports scientific research based on Real-World Data (RWD), verified by medical specialists using evidence-based medicine (EBM). The platform covers 10 clinical situations. The applied method was illustrated using one situation—a breast X-ray examination—employing clinically approved structures and real-world validation. Approved by the Bioethics Committee, the system is currently being tested at the hospital, marking a significant step toward efficient, reliable, and sustainable healthcare documentation.

## Introduction

Physicians’ well-being is crucial to maintaining high-quality healthcare, as excessive workloads can lead to stress, job dissatisfaction, and, ultimately, burnout among healthcare providers^1^. This not only affects the individual health and well-being of physicians but also harms the quality of healthcare services. To address this issue, creating a supportive work environment that alleviates physicians’ workload is essential^2,3^. To combat burnout, a multifaceted strategy is necessary that provides for organizational reforms, targeted interventions in specific situations, and adjustments to the organization of work. The recommendations focus on reducing administrative burdens, creating a supportive work environment, and implementing health promotion programs that address both systemic and individual stressors^4^. The solution we have developed aims to create a working environment that supports doctors in their daily duties by reducing their workload when creating EHRs and limiting the potential for errors by verifying the completeness of the documentation.

Recognizing a doctor’s speech in natural language and interpreting it correctly has enormous potential to create accurate and complete Patient EHRs, improve the quality of medical information about patients, and enable future scientific research on large data sets based on RWD^5^.

In the field of modern emergency medicine, one of the elements of the presented project, artificial intelligence (AI), is being increasingly used to enhance patient management, improve care effectiveness, and support clinical decision-making in environments that require immediate responses. Breakthrough innovations encompass not only classic Clinical Decision Support Systems (CDSS) and EHRs, but also the integration of Emergency Department Information Systems (EDIS) and Ambient Clinical Intelligence (ACI) solutions^6,7^. Adaptive intelligent speech processing systems (AISPS), which enable real-time adaptive speech recognition and analysis, remain an emerging area of study, offering the potential to automate communication and documentation at the most critical moments of medical care, such as cardiopulmonary resuscitation or resuscitation team activities^8,9^.

## Background

Voice-enabled EHR systems, ambient scribing pipelines, and LLM-based text-to-FHIR (Fast Healthcare Interoperability Resources) frameworks reported to date are predominantly developed and evaluated in English and a few high-resource languages, combining modern Automatic Speech Recognition (ASR) with medical Named Entity Recognition (NER), concept normalization, and structured note generation or direct FHIR resource synthesis^10,11^. Typical evaluations of voice-enabled or text-based clinical Natural Language Processing (NLP) systems report ASR WER (Word Error Rate) and classification-oriented metrics such as precision, recall and F1, sometimes complemented by confusion matrices, while FHIR-conversion frameworks additionally employ measures such as failure rate, hallucination rate and resource mapping accuracy^12,13^. In contrast, Polish-language studies are fewer and generally scoped to narrow sub-tasks (e.g., dictionary-aided recognition, domain lexicons, or small-scale corpora), reinforcing the need for language-specific resources and evaluation protocols^14,15^. Our work differs by operationalizing ten predefined clinical situations with transparent ontologies and rule-based completeness checks, coupling ASR —> grammatical error correction (GEC) —> NER with situation-specific slot filling to produce structured EHR entries ready for integration in this-work. Together, these design choices target context fidelity and documentation completeness for Polish clinical workflows.

The key to a complete EHR record is accurate speech recognition, followed by the correct conversion of speech to text, and then a thorough understanding of the context of the recorded information. In English, this task is already being performed with great success by many LLMs, but in less-resourced national languages correct context recognition remains a significant challenge. This difficulty is well documented for clinical NLP/ASR outside English, where limited annotated corpora, scarce medical lexicons, and frequent code-switching with Latin/English terms impede context-sensitive modeling and downstream extraction quality^12,16^. For Polish in particular, public domain-specific resources have only recently begun to emerge, and general-purpose models underperform without language- and domain-adaptation^14,15^. Due to the limited number of data sources, creating complete repositories for specific medical applications in national languages is an additional challenge. In Poland, EHRs have been widely available in electronic form only for a few years, and in many medical facilities a significant portion of documentation is still maintained on paper. The Polish medical register also mixes Polish with Latin and English terminology and uses numerous abbreviations, further complicating normalization and disambiguation^17^. Consequently, the complexity of records and scarcity of digitized resources pose a challenge for LLM training and evaluation in unambiguously identifying and interpreting Polish medical records. Therefore, we developed specialized, situation-focused tools tailored to Polish clinical contexts.

## Methods

As a result of a project carried out at Gdańsk University of Technology in cooperation with Gdańsk Medical University, a solution is being developed and implemented that enables doctors to create dedicated EHRs using voice commands. This involves filling in selected patient medical records in and prescribing treatment as needed^18^. We have precisely defined the range of clinical situations in which the language model will be a valuable tool to support doctors in creating EHRs. To do this, we selected 10 clinical situations, described their ontologies, designed the structure of the complete medical information necessary to be collected in the EHR documenting clinical situations, gathered the appropriate training sets, conducted tests of solutions, selected the most effective solutions, and prepared applications along with the proper equipment for use by doctors in their daily clinical work.

Clinician involvement was integrated throughout the early development phase of the system. Practicing physicians, nurses and technicians participated in defining the clinical scenarios in which an adaptive speech-driven EHR assistant would provide the greatest value (e.g., emergency medicine, cardiology, surgery). They also contributed to specifying the structure and key elements of medical documentation for each scenario, ensuring alignment with real-world workflows. Furthermore, clinicians assisted in assembling representative textual material used for training and evaluating the system. In the next stage of development, we plan to conduct structured validation sessions with a broader group of practitioners to assess usability, accuracy, and completeness of generated documentation. Their feedback will directly guide refinements of the speech-recognition pipeline and the context-analysis modules.

Our goal was to create an EHR creation tool for doctors that leverages AI and human expertise to complement each other. AI correctly structures the collected information, assigns it to the appropriate categories, and, thanks to algorithmization, indicates the required additions. Algorithmization, for the designed forms, means calculating selected numerical values in places required by the description of the clinical situation, and marking and informing the user about missing data in order to ensure the completeness of the description.

The application uses the obtained, structured medical data to develop a situation analysis on the basis of EBM. The EHR documentation created includes the necessary information required for assessing the patient’s condition.

Our AI platform is designed to maintain stability through continuous performance monitoring and periodic updates to its speech recognition models, ensuring reliable operation in dynamic clinical settings. It demonstrates robustness by adapting to diverse speech inputs, including regional accents and complex Polish medical terminology, and is validated across various hospital environments. Furthermore, its sustainability is supported by seamless integration with existing(EHR systems and a scalable architecture that can evolve with changing healthcare demands. In the long term, it enables the observation of large groups of patients over extended periods, thanks to the consistency and completeness of the observed parameters. The application is currently in Technology Readiness Level (TRL) phase 7, testing the proof-of-concept (POC) for each medical situation. In 6 months the application will have reached TRL 9 and will be launched. Finally, although this study focused solely on text-based reports, our larger research project includes audio recordings of physician–patient interactions, which will eventually require robust speech-to-text pipelines. The results of the project work emphasize the need to develop a tool capable of recognizing Polish medical terms. Based on English translations, the results obtained during this work confirm the thesis that such a tool must be developed and thoroughly tested to achieve even greater reliability. Currently, there is no Polish EHRs.

### Materials

This work aims to create an application that generates medical documentation during doctor-performed medical procedures. Therefore, the topic of developing the appropriate form of documentation and methods for recording and then structuring text for EHR by clinical needs was taken up. This work was created as part of the project "Adaptive System for Intelligent Speech Processing by Doctors," which includes structuring test results and supporting the therapeutic process, and is financed by the EU’s Infostrateg program and the National Centre for Research and Development in Poland.

Due to the specific nature of the Polish medical language, used to describe a medical situation precisely, the work was divided into 10 situations in 3 groups:

1. Situations in a controlled environment. Working with an object—a sample: description of a radiological examination, description of a pathomorphological examination. These are situations corresponding to controlled acoustic conditions in which the recording is made (e.g., an office or a laboratory).
2. Situations with complex conditions. Working with a patient involves conducting a medical interview, performing an oncological examination, and a cardiological examination, providing recommendations, referring to the patient for treatment, and issuing a prescription. The increased complexity of recording in a clinical setting results from environmental acoustics, personal diversity, and room complexity, as well as ambient noise.
3. Highly complicated situations. Dynamic situations: surgical procedure – perioperative control card, resuscitation protocol. The high degree of complexity stems from the dynamics of events, the large number of participants, the use of protective masks by participants (including doctors and medical staff), and noise interference from medical equipment.

### Clinical situations and data collection

The clinical situations we have analysed and for which we are developing solutions are as follows:

- conducting a medical interview,
- performing an oncological examination,
- performing a cardiological examination,
- description of a radiological examination
- description of a pathomorphological examination
- providing recommendations,
- referring to the patient for treatment,
- issuing a prescription
- surgical procedure—preparation of perioperative control card,
- performing a resuscitation protocol.

The language model training existing texts obtained with the help of Hospital Information System (HIS) data extraction, which was performed using the MedStream Designer (MSD) tool. The texts were obtained from patient records (EHRs) collected in the HIS of the University Clinical Center (UCC) in Gdańsk. The records were selected according to clinical situations and completely anonymized and divided into shorter, several dozen-word fragments to make them easier to read. The ranges of retrieved texts were described using scenarios. The following were obtained sequentially for each clinical category:

- medical history: 1520 texts
- oncology outcome: 2221 texts
- radiological examination result: 28,870 texts
- pathomorphological examination result: 1592 texts
- result of cardiology examination: 1729 texts
- course of surgery: 33,947 texts
- resuscitation scenario: 20 scenarios and 1962 texts
- medical orders: 2788 texts
- referral: 1000 texts
- prescribing: database of 127,081 drug names and descriptions

The resulting database enabled the experiments to be carried out effectively; however, in some cases, it was necessary to expand the database with dedicated dictionaries.

Throughout the project, a database of 75,651 source texts and a database of 127,000 drug names with descriptions were compiled, based on which 114,354 text recordings for 166 individuals (voices) were recorded. Due to the project’s vast scope, the publication presents the method of work using the example of a chosen area of research: the extraction and classification of medical information from a doctor’s spoken text describing a breast echocardiography examination in cases of suspected breast cancer.

For this type of study, 3,841 recordings were made, which were then transcribed and analyzed in the manner described in the paper. The detailed information about used dataset were presented in the paper^14^.They were then transcribed and placed in context using a machine learning system trained through knowledge distillation, consisting of Llama3.1-70B-Instruct as the teacher model and distilbert-base-multilingual-cased as the student model^19^. Knowledge distillation is a model-compression technique in which a smaller “student” model learns to mimic a larger “teacher” model—usually by copying its soft output distributions (logits) and possibly its internal representations.

### Speech recognition and ues

**Fig. 1:**
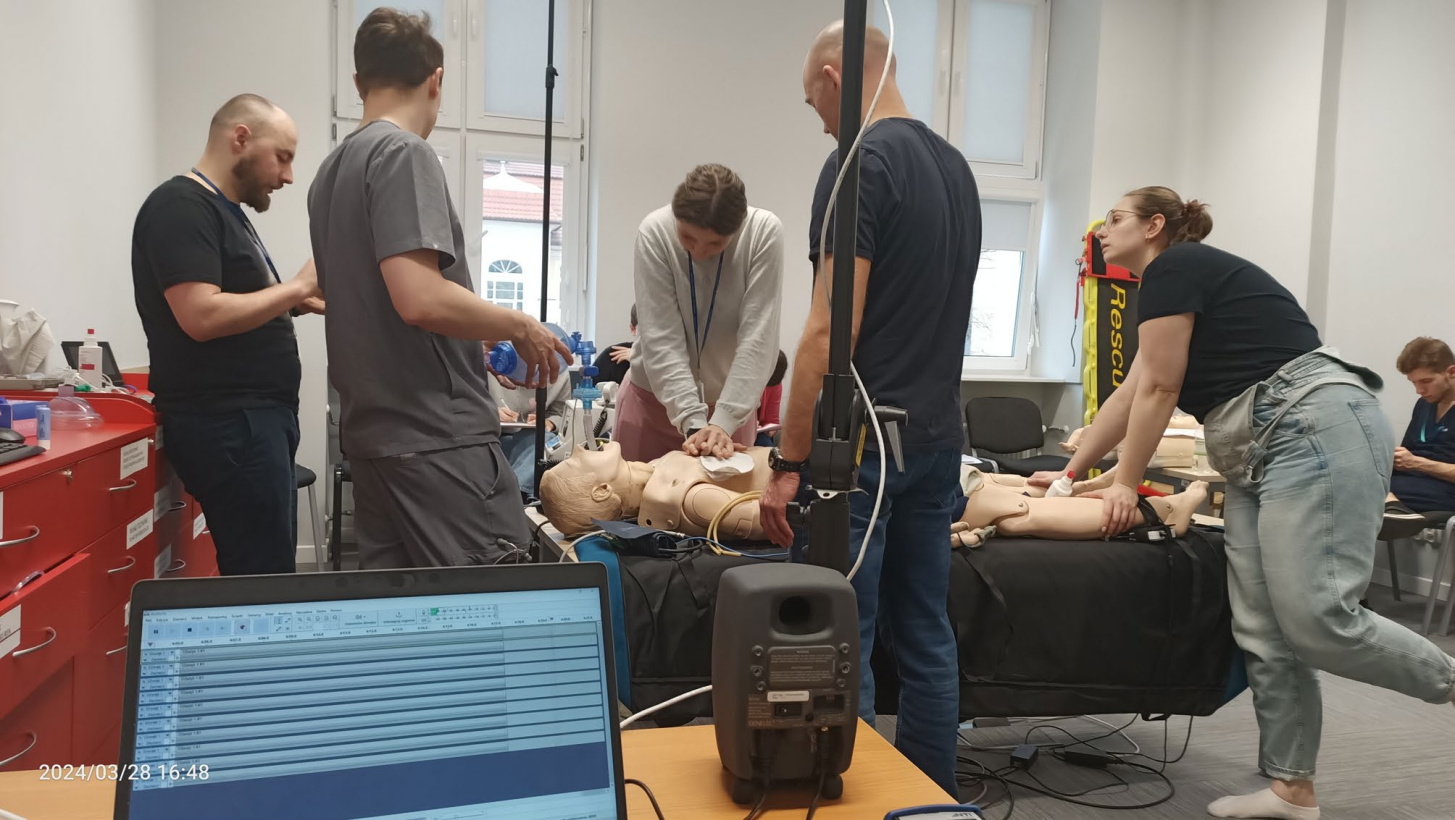
Simulation and training of resuscitation to prepare a resuscitation protocol and record a soundtrack.

After a phase of test recordings and analysis of texts from actual patient documentation, we created a real environment for data collection and continued further testing. To ensure proper sound recording, a special microphone was developed at the Department of Multimedia Systems at Gdańsk University of Technology – a vector acoustic sensor, called an acoustic probe. The acoustic probe and speech recognition algorithms are calibrated to ensure stable performance across a wide range of hospital acoustics, from quiet offices to noisy operating theaters. The detailed information about the sensor and its calibration process can be found in the paper^20^ Robustness is enhanced by advanced error correction techniques, such as MaskGEC^21^, which dynamically adapts to transcription errors, and Drug-NER^22^, which accurately identifies complex medical terms. For sustainability, the system supports ongoing updates to its medical dictionaries and algorithms, ensuring long-term relevance as clinical practices evolve (Fig. 1).

The most challenging task was recording and interpreting the resuscitation actions. To structure the information from the resuscitation action, a special protocol for communication among the resuscitation team was created, which aligns with the standards and best practices established by the European Resuscitation Council (ERC). To train the model, 80 h of recordings of simulated resuscitation actions in 20 different scenarios were conducted, followed by the preparation of dictionaries of commands, messages, and drugs used to document the course of the rescue action. The methods described in this publication were used to train the model to recognize the content of communication. The recording protocol was extended to capture precise timestamps for messages deemed critical to the resuscitation. Messages were collected into a separate dictionary, which the text recognition model overwrote.

#### The use of error correction methods in speech recognition

In order to correctly transcribe the recognized text, we tested several methods of linguistic error correction. In the first phase, to describe clinical situations, dictionary-based techniques were used to correct errors and ensure clarity in diagnoses. The basic principle of these methods is to compare words in the text with a database of correct words, such as a dictionary. These algorithms check whether a given word is in the dictionary and, if not, suggest the most likely corrections based on similarity measures, such as the Levenshtein distance or fuzzy matching. Fuzzy matching is a technique for finding words that are similar to misspelled words, though not necessarily identical. Fuzzy matching algorithms use edit distance metrics to calculate the distance between character strings. The most popular distance metrics include Levenshtein distance, Damerau-Levenshtein distance, and Hamming distance. These metrics were tested. To apply fuzzy matching, keyword lists specific to the features sought in the clinical situation description were created. The Jaro-Winkler and Soundex algorithms were tested, which are widely used to recognize errors resulting from similar-sounding words. This is particularly useful when searching for names and in specialist terminology, where minor differences in spelling often occur.

#### The effectiveness of dictionary methods in medical vocabulary

In specialized fields such as medicine, dictionary methods may not produce satisfactory results. The dictionary algorithms described above are good at detecting typos and simple spelling mistakes, but they may have limitations when dealing with specialized terminology. Medical vocabulary is characterized by a large number of technical terms that are often long and complex, with specific suffixes and prefixes. Therefore, if a particular term is missing from the dictionary, dictionary algorithms may not find suitable suggestions. It was therefore crucial to develop rich and comprehensive dictionaries for each clinical situation. The dictionaries were constructed from actual EHR texts, official disease classifications, and ICD-9 and ICD-10 procedures. Specialists in the field supplemented them. The challenge remains to keep the terminology current. In medicine, new terms often appear, related to technological advances, new therapies, or drugs. Dictionaries must be regularly updated to include new words.

To enhance the effectiveness of language error correction in texts that contain medical terminology, dictionary methods have been supplemented with context analysis (described below). Combining dictionaries with a language model that analyzes sentence context enables more precise matching. Thanks to this, the model can identify that, for example, ‘amoxicillin’ is more appropriate for treating bacterial infections than ‘ampicillin’, even though both words are similar.

#### Methods based on deep learning

The next phase of testing was the application of deep learning, which is characterized by the model learning from large datasets, assimilating patterns, relations, and word-to-sentence relationships. Unfortunately, in our clinical situations, the test sets proved too small and too schematic. Details about the database size can be found in the previous section. The texts in the database were derived from the HIS description templates doctors used to speed up descriptions. Classical Recurrent Neural Networks (RNNs), Long Short-Term Memory (LSTM) networks, and Seq2seq models were tested. However, their effectiveness on the existing text database was too low (high Word Error Rate—WER), so context-enhanced dictionary methods were used for correction. WER is defined according to formula:

$$WER = \frac{S + D + I}{N} \times 100\%$$

where *S* – Substitutions (words incorrectly recognized, e.g., “cough” → “coffee”); *D*– Deletions (words omitted from transcription); *I* – Insertions (extra words added); *N* – Total words in reference transcript.

According to literature, non-trained- Whisper achieved 20.84% WER on Polish medical conversations and this value is not practically acceptable^15^. Also according to literature, for English medical language, WER below 10.5% is considered as state-of-the-art^23^.

One significant challenge in analyzing doctors’ speech with transcription models, such as Whisper, is accurately recognizing drug names. Despite its high performance in general tasks, Whisper encounters problems with medical terminology (especially in Latin).

The project, therefore, turned its attention to Drug Name Recognition Technology (Drug-NER). Drug-NER is a natural language processing task that focuses on identifying and classifying drug-related terms in medical texts.

The Drug Named Entity Recognition library in Python was used to perform this task^22^. The testate data came from the Drugbank database, augmented with datasets from the NHS, MeSH, Medline Plus and Wikipedia in a cell object dictionary of broad medical terminology. The dictionary also included disease names.

At the outset, the model applies a classification layer to identify names as entities, assigning each detected phrase an appropriate category, e.g., drug, active substance or disease. The library enables flexible matching. As part of the system’s testing, a study was conducted on the ADMEDVOICE database^24^. 45 recorded records were retrieved, containing the names of drugs, active substances, and diseases (e.g., *I prescribe Valsartan Medical Valley tabl. 4 packs of the product of 14 tablets*). The model correctly recognized 42 of these, giving a success rate of 93%. Local Polish drug substitutes were not identified. The database was enriched with a drug dictionary derived from the hospital’s local drug database. To be fully effective, the system still needs to be tested with additional dictionaries containing different types of abbreviations and units of measurement. Work and these dictionaries are ongoing.

#### Correcting words using NLP techniques

To improve speech-to-text transcription quality, research was conducted on adding methods for correcting words using NLP techniques, leveraging models from the Whisper family^25^. The application of NLP methods can significantly improve transcription quality by eliminating potential errors^26^. In further research, we focused on to handle better machine learning methods that adapt to specific errors generated by speech recognition systems (ASR), introducing a dynamic input masking technique for grammatical correction models that allows the system to handle typical ASR errors^21^ better. This method, referred to as MaskGEC, enhances correction accuracy and facilitates a better fit between the transcription and the task. An additional advantage of using NLP is that the exact solutions can be applied to different languages without significant changes^27^. This is useful for languages with limited resources (amount of available data) and difficult, ambiguous pronunciation (e.g. Polish). Furthermore, numerous studies emphasize that NLP is extremely useful in a medical context, which, together with the challenge of working in Polish, contributes to the difficulty and specificity of the project^28–30^. NLP enables the recognition and interpretation of medical terminology, which is essential in analyzing clinical data, especially in the context of EHRs^30^.

### Methods—ethical and data protection statement

All clinical data used in this study consisted of previously collected medical records, including clinical notes, pathology reports, surgical descriptions, and diagnostic imaging reports. Prior to analysis, all materials were fully de-identified by removing personal identifiers and any information that could allow re-identification of individual patients. No new medical interventions, recordings, or additional diagnostic procedures were performed for the purposes of this project. The study therefore had no direct or indirect impact on the clinical care of the patients whose historical documentation was used. The use of de-identified data and the overall study protocol were reviewed and approved by the University Bioethics Committee, which confirmed that the procedures met ethical and data-protection standards.

## Results

The goal of our work was to develop an application that would effectively and accurately handle EHR forms. Work on the application was carried out during the registration and training of the speech recognition and context recognition model in accordance with EHR requirements.

The application sends audio in real time to a model that transcribes speech to text. Then, using a language model with overarching, dedicated vocabularies, the text is split into contexts that correspond to the form’s link. If inference is performed based on the provided data, the clinical situation-specific algorithmizing is applied to the result when the form is completed. The completed form is accepted or corrected by the clinician, saved as an EHR, and sent to the HIS.

It is essential to note that components of the AI ecosystem, such as ACI and English-language ambient scribe systems, are rapidly approaching implementation maturity. These platforms can automatically capture clinical conversations and interactions, convert them into structured electronic records, and integrate them with electronic medical records, thereby reducing staff administrative burden and allowing them to focus on direct patient care^31–33^. Studies have shown that the use of ambient scribes substantially reduces the time required for documentation preparation and improves the quality of documentation^31,32^.

Meanwhile, reducing burnout among emergency department physicians has been identified as an additional benefit of these technologies^33^. Furthermore, automated AI platforms for documentation have been positively evaluated by clinicians for usability and may contribute to increased job satisfaction^10^, as discussed in the introduction.

### Data collection outcomes

The result of working with data obtained from both the UCK EHR database and data recorded during the simulation is the correct recognition and conversion of speech into text, followed by recognition of the context in which the text was created. On this basis, it will be recorded in the appropriate field of the EHR form. A context recognition analysis was performed for each of the 10 clinical situations. A proper tagging system was created to accurately describe the clinical situation based on evidence-based medicine (EBM). An interactive form was designed, and the necessary algorithms were implemented within the application. To the context for each clinical situation. Due to the large amount of material, we describe how we developed the methods in this publication using one selected clinical situation as an example.

In this study, we focus on extracting information from radiology reports in the context of breast imaging. These reports usually contain descriptions of breast tissue, specific findings, measurements, BIRADS categories ((a standardized breast imaging rating system)^34^, lymph node evaluations, and clinical recommendations. However, this information is typically embedded in an unstructured free-text format, making it difficult to extract and analyze systematically.

### Context recognition results for breast radiology examination

A challenge in processing radiology reports is that information of different types is often presented together within the same narrative. To enable structured analysis, it is necessary to segment the text into coherent fragments and classify them into predefined categories. In this study, we address this challenge by dividing reports into smaller textual units—based on paragraph boundaries—and applying a classification approach to assign each fragment to a relevant label^2,4,6–9,18,26,27,31–33,35–37^.

To support this task, we used BERTopic, a topic modeling technique that combines transformer-based embeddings with clustering. Unlike traditional models such as Latent Dirichlet Allocation (LDA), which rely on shallow statistical features (e.g., TF-IDF), BERTopic captures deeper semantic relationships between words and phrases, making it better suited to the nuanced language of medical texts^38^. Although BERTopic is primarily designed for unsupervised topic modeling, we adapted it for use in a classification context, aligning its generated topics with a set of predefined labels curated by clinicians^39,40^

Our motivation for exploring BERTopic standard common limitations in clinical NLP applications. We also examined whether this approach could suggest a meaningful structure for radiology reports, which often follow implicit conventions rather than rigid templates. Our findings indicate that BERTopic can extract clinically relevant groupings that align with expert-defined categories, even if it does not capture finer distinctions (such as left vs. right breast) that require broader contextual awareness.

The trained model was then applied to a breast radiology examination form designed with clinical requirements. Forms of this type were prepared for each clinical situation.

Breast radiology reports, while often loosely structured, typically follow implicit patterns that can be broken down into distinct informational components. In our study, we leveraged this characteristic by segmenting entire reports into smaller, coherent fragments, based on the original paragraph structure. This process was applied to a dataset of 6,269 breast ultrasound reports sourced from real hospital documentation, yielding 48,721 text fragments. These fragments served as the training data for our topic modeling algorithm.

### Results of BERTopic approach

**Fig. 2:**
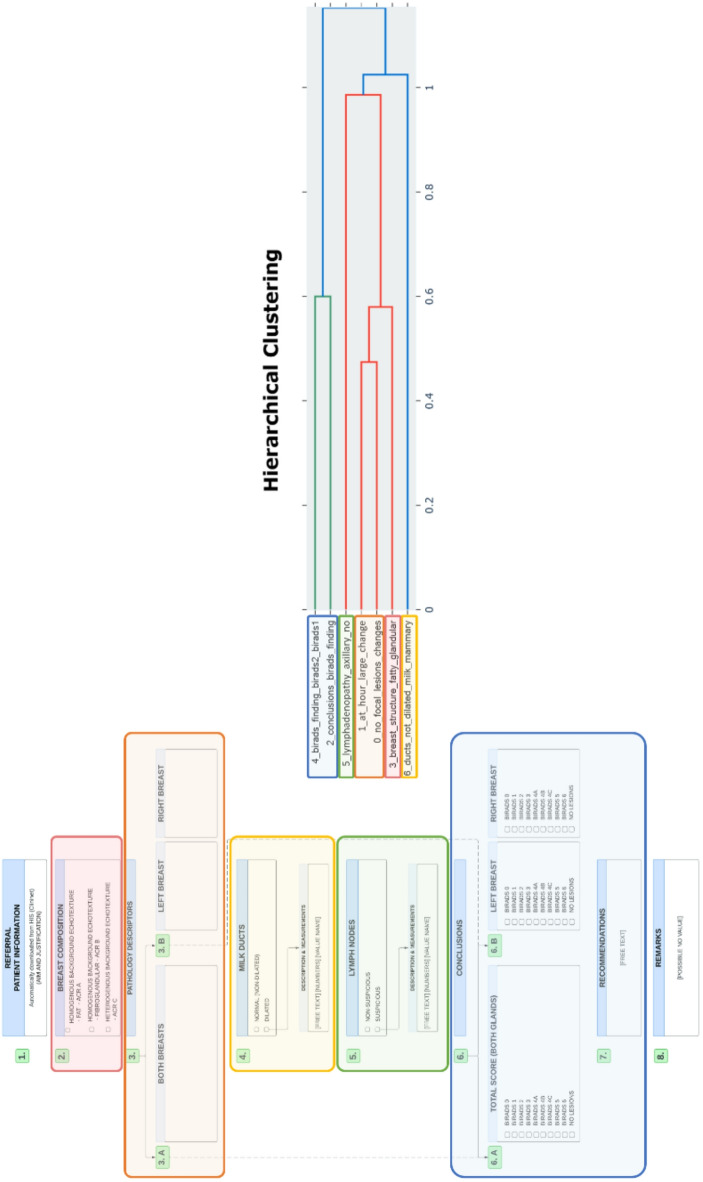
Defined by clinicians’ structure of breast radiology reports (left), BERTopic extracted topics (right).

To assess our approach, we used a target structure for breast ultrasound reports defined by our clinical experts, consisting of eight key informational categories: referral data (sourced from HIS system), breast structure, breast description, milk ducts assessment, lymph node assessment, BIRADS classification, recommendations, and other or miscellaneous content. This expert-defined structure, shown in Fig. 2, serves as a post hoc reference for evaluating the outputs of the topic modeling process.

Notably, we deliberately excluded any human-validated labels from the training phase to maintain a fully unsupervised setting. This design choice reflects a real-world scenario in which labeled data is limited or unavailable, common in many clinical and medical text domains. By evaluating the extracted topics against a separate, manually annotated dataset by our clinicians, only after training, we aimed to assess whether an unsupervised model like BERTopic can still recover a meaningful structure from unstructured clinical texts^41^. Data was annotated using Label Studio^42^.

To model the semantic structure of the text fragments, we configured a BERTopic pipeline. For generating embeddings, we used the paraphrase-multilingual-MiniLM-L12-v2 model from the Sentence Transformers library^43^, which is well-suited for multilingual data and performs efficiently even on relatively short text segments.

Dimensionality reduction was applied to the resulting embeddings using Principal Component Analysis (PCA), reducing the vector space to three dimensions. This step facilitates more effective clustering by eliminating noise and focusing on the most informative features. Subsequently, we used K-Means clustering to group the fragments into topics. The number of clusters was set to 7, reflecting the number of categories defined by our domain experts (excluding the referral section, which is not part of the image test itself).

This choice enabled us to assess how well the learned topics aligned with the known components of breast ultrasound report structure. As shown in Fig. 2, the extracted topics (right) were color-coded to reflect their alignment with the reference structure. The results revealed some degree of overlap, with specific topic issues—clusters 0 and 1, as well as 2 and 4—frequently merging into single sections. While clusters 0 and 1 both fall under the clinician-defined breast description category, closer inspection reveals that they are semantically distinct. Cluster 0 primarily captures descriptive findings, whereas cluster 1 tends to reflect measurements and the localization of those findings. Similarly, clusters 2 and 4 can be mainly mapped to the conclusion section of the report. However, they also contain elements related to recommendations, indicating some thematic overlap between these two categories, which is reasonable as these sections are often written in the same paragraph boundary in a breast ultrasound report.

The BERTopic-based approach demonstrated promising alignment with the structure proposed by clinical experts, successfully identifying key sections of the breast ultrasound reports that correspond to the main headers used by radiologists.

Despite being trained in a fully unsupervised setting—without any access to labeled data during training—the model was able to extract topics that closely reflect the intended informational structure of the reports.

**Fig. 3:**
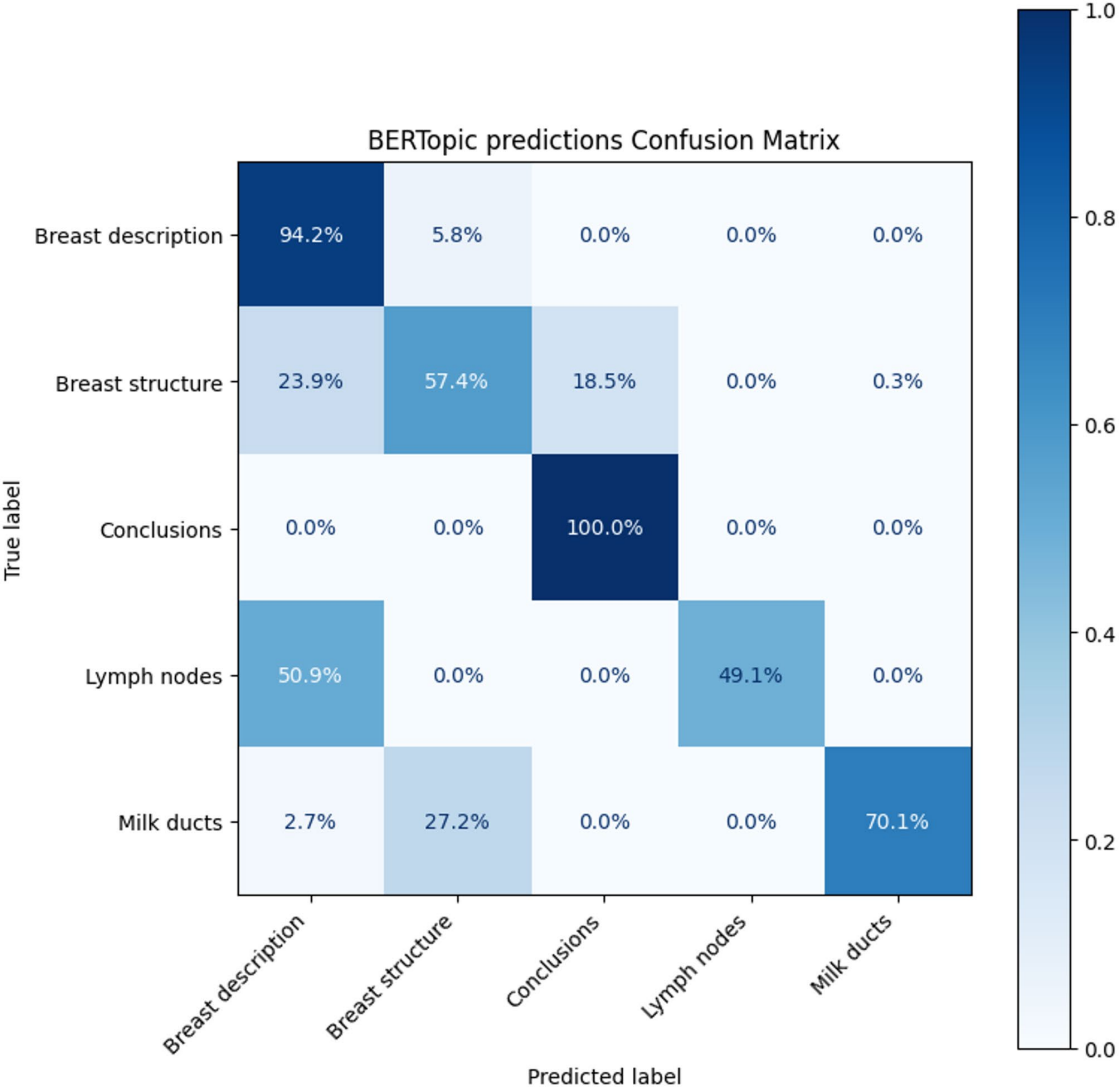
Normalized confusion matrix for BERTopic model predictions.

The validation data consisted of 1,111 clinician-validated text fragment-label pairs, sourced from breast ultrasound reports written by various practicing physicians. During validation, the model achieved an average accuracy of 73% across all predefined labels. A closer look at the confusion matrix (Fig. 3) reveals particularly strong performance in certain categories:

- Breast description with 94.2% recall, 54% precision, 68,5 F1-score (223 samples),
- Conclusions with 100% recall, 74,3% precision, 85,3 F1-score (199 samples),
- Milk ducts with 70.1% recall, 98,9% precision, 82,1 F1-score (147 samples),
- Breast structure with 57.4% recall, 80,2% precision, 66,9% F1-score (373 samples),
- Lymph nodes with 49.1% recall, 100% precision, 64,9% F1-score (169 samples).

Work with content classification using BERTTopic has enabled the development of a classifier. The results support the feasibility of using BERTopic as a lightweight, unsupervised classification tool for structuring clinical text, including Polish-language texts. Its performance is auspicious in scenarios where labeled training data is unavailable or difficult to obtain, making it a potentially valuable tool for exploratory structuring of other types of medical narratives.

To improve doctors’ speech recognition quality, it is beneficial to use native-language models. Research on applying large language models to improve ASR accuracy is a rapidly growing field^21^. That study focuses on using LLMs for error correction by analyzing multiple hypotheses generated by ASR (N-best outputs). The authors employed various approaches, including zero-shot and one-shot learning, to effectively reduce errors and enhance the comprehensibility of the generated transcriptions. We are testing the Polish language model Bielik for this purpose. The Bielik model, an advanced Polish language model, has particular potential as a language corrector for large systems, such as the Whisper model. Based on the experience of the work presented above, it was hypothesized that the integration of Bielik with Whisper would bring benefits in areas such as the correction of spelling errors, Correction of typos, and Correction of inflectional errors. We can use this correction in further work.

In the next steps, we plan to continue exploring supervised approaches and further develop the topic modeling method using additional data to compare the effectiveness of both strategies. While the topic modeling approach demonstrated promising performance in recovering domain-relevant categories, it also revealed essential limitations. First, the BERTopic model does not account for global contextual information within reports. As a result, it is unable to differentiate clinically meaningful nuances such as laterality (e.g., left vs. right breast), which often requires document-level context or co-reference resolution. Second, some semantically adjacent categories (e.g., conclusions vs. recommendations) were partially merged by the model, reflecting the fact that these elements are often written in shared paragraphs and lack explicit boundaries.

## Current status and validation

The current status of the work is to test continuous tests of application interfaces for each of the 10 described clinical situations under real-world conditions. Acoustic probes have been installed in doctors’ rooms; a test probe has also been installed in the operating theatre and in the Shock Room of the Hospital Emergency Department, where resuscitation is carried out. The accuracy of context recognition is improved using the methods described above. A method for creating clinical accuracy benchmarks for recognized and transcribed texts is established. The application are being improved. Real-world testing across 10 clinical situations demonstrated the system’s stability, with consistent speech recognition accuracy (e.g., 93% for drug names) over prolonged use in hospital settings. Its robustness was validated in diverse scenarios, ranging from controlled radiology labs to high-stress resuscitation rooms, where it adapted to noise and dynamic interactions. Sustainability is ensured through compatibility with Hospital Information Systems (HIS), allowing seamless data exchange and updates without interrupting clinical operations.

## Discussion

The results of the project work emphasize the need to develop a tool capable of recognizing Polish medical terms. Based on English translations, the results obtained during this work confirm the thesis that such a tool must be developed and thoroughly tested to achieve even greater reliability.

It is essential to integrate all systems that utilize AI to record and interpret doctors’ speech with the Electronic Health Record (EHR), as this is particularly crucial in stressful situations, such as those occurring in the Emergency Department. Such integration not only enables rapid, secure collection and analysis of clinical data but also creates the conditions for implementing increasingly advanced tools to support clinical decisions. The key to effective AI use in medicine is not only the quality of the algorithms, but also the ergonomics of their implementation, the smoothness of their integration with existing infrastructure, and the readiness of staff to adapt to new digital solutions^6,7^. It has been emphasized that, for effective teamwork, it is imperative to design systems that support time management, task coordination, and transparent communication, which requires close collaboration between IT and emergency medicine professionals^7^.

Involving clinicians throughout the design and evaluation of health-information technologies is widely recognized as critical for usability, workflow integration and adoption. Our approach, in which practitioners helped to define high-value clinical scenarios, specify documentation structures across specialties, and contribute representative texts, follows principles of user-centred and participatory design shown to improve clinical decision support and guideline-based systems^44–46^. This is particularly important for speech-driven documentation tools, where prior work has highlighted both the potential benefits and risks of speech recognition for clinical documentation, including its impact on efficiency, safety and error patterns^47,48^. Our planned next step—structured validation sessions with doctors using the deployed system—is consistent with surveys indicating that clinicians’ real-world experience, perceived workload and satisfaction strongly shape the successful uptake of speech-based EHR solutions^49^.

## Conclusions

In summary, although artificial intelligence is increasingly entering medical practice, and the implementation of tools such as Ambient Scribe and automatic documentation brings measurable benefits, there is still a lack of reliable research on the effectiveness and safety of Adaptive Intelligent Speech Processing Systems. Further research—particularly randomized clinical trials in real-world settings—is essential to confirm the efficacy and determine the optimal conditions for implementing these innovative solutions^2,8,9^.

In our study, we observed significant variation in the process of annotating medical terms among physicians from different specialties. This diversity of annotation practices underscores the complexity of medical language and highlights the unique perspectives that different medical disciplines bring to the interpretation of terminology. Our findings suggest that the interdisciplinary knowledge inherent to the medical profession significantly shapes the categorization of medical terminology, underscoring the need for tailored natural language processing (NLP) solutions that account for subtle differences across medical fields. This knowledge not only enriches our understanding of medical language processing but also underscores the importance of developing flexible, specialized NLP tools that support the diverse needs of the healthcare sector.

Applying statistical analysis to the results of our experiments showed that the effectiveness of AI tools and doctors in annotating medical terms is statistically indistinguishable across various categories, including drugs, diseases, and symptoms, procedures, and other medical terms. This equivalence of results challenges the assumption that either AI or human expertise is superior in annotating medical texts. Instead, it highlights the potential of a synergistic approach in which AI tools complement the subtle understanding of medical specialists. This integration could potentially lead to more accurate and comprehensive annotations, improving the quality of medical databases and supporting the development of advanced AI applications in healthcare.

To ensure and enhance stability of the solution, we have implemented automated performance monitoring tools to detect and address degradation in real time. Robustness will be further tested in high-pressure settings, such as emergency departments, and with diverse user groups, including non-native speakers. To ensure sustainability, we plan to establish a user community that contributes to dictionary updates and develops a scalable framework for adapting the system to additional clinical domains.

In conclusion, our AI platform advances real-world healthcare by reducing physicians’ administrative burden while ensuring stability through consistent performance in clinical settings, robustness via adaptive error correction and context recognition, and sustainability through integration with existing systems and scalability for future needs. This positions it as a practical, enduring solution for modern healthcare documentation.

## Data Availability

The anonymized datasets generated and analyzed during the current study are available from the corresponding author upon reasonable request.
